# Plate fixation of inferior ramus in pubis-ischium ramus improves mechanical stability in Tile B pelvic injures: a cadaveric biomechanical analysis and early clinical experience

**DOI:** 10.1186/s12938-024-01262-8

**Published:** 2024-07-12

**Authors:** Zhongjie Pan, Lili Qin, Xiaorong Shi, Feng Hu, Yuquan Li, Muwen Li, Min Chen, Wengui Huang, Yuanjun Li, Zhi Yang, Jinmin Zhao, Wei Liu

**Affiliations:** 1 Department of Orthopedic Trauma & Hand and Foot Surgery, The Second Affiliated Hospital of Guangxi Medical University, Nanning, Guangxi China; 2 Department of Trauma Surgery, The Second Affiliated Hospital of Guangxi Medical University, Nanning, Guangxi China; 3 Department of Orthopaedics Trauma and Hand Surgery, The First Affiliated Hospital of Guangxi Medical University, Nanning, Guangxi China; 4 Department of Orthopaedic Joint Surgery and Sports Medicine, The Second Affiliated Hospital of Guangxi Medical University, Nanning, Guangxi China; 5 Department of Orthopedics, The Peoples Hospital of Yudu County of Jiangxi Province, Ganzhou, Jiangxi China

**Keywords:** Inferior ramus, Pubis-ischium ramus, Tile B pelvic injuries, Biomechanical, Plate fixation

## Abstract

**Background:**

Management of inferior ramus of the pubis-ischium ramus remains controversial, and related research is sparse. The main intention of this study is to describe the biomechanical and clinical outcomes of pubis-ischium ramus fractures in Tile B pelvic injuries and to identify the feasibility and necessity of fixation of the inferior ramus of the pubis-ischium ramus.

**Methods:**

This study comprised two parts: a biomechanical test and a retrospective clinical study. For the biomechanical tests, Tile B-type pelvic injuries were modeled in six cadaver specimens by performing pubis-ischium osteotomies and disruption of the anterior and interosseous sacroiliac ligaments. The superior and/or inferior rami of the pubis-ischium ramus were repaired with reconstruction plates and separated into three groups (A, B, and C). Specimens were placed in the standing position and were loaded axially with two-leg support for three cycles at 500 N. The displacements of sacroiliac joints at osteotomy were measured with Vernier calipers and compared using statistical software. To investigate the clinical outcomes of this technique, 26 patients were retrospectively analyzed and divided into a superior ramus fixation group (Group D) and a combined superior and inferior ramus of the pubis-ischium ramus fixation group (Group E). The main outcome measures were time of operation, blood loss, postoperative radiographic reduction grading, and functional outcomes.

**Results:**

In the vertical loading test, Group E showed better pelvic ring stability than Group D (*P* < 0.05). However, the shift of the sacroiliac joints was almost identical among the three groups. In our clinical case series, all fractures in Group E achieved bony union. Group E demonstrated earlier weight-bearing functional exercise (2.54 ± 1.45 vs 4.77 ± 2.09; *P* = 0.004), earlier bony union (13.23 ± 2.89 vs 16.55 ± 3.11; *P* = 0.013), and better functional outcomes (89.77 ± 7.27 vs 82.38 ± 8.81; *P* = 0.028) than Group D. The incidence of sexual dysfunction was significantly lower in Group E than that in Group D (2/13 vs 7/13; *P* = 0.039). Bone nonunion occurred in two patients in Group D, and two patients in Group E had heterotopic ossification. None of the patients exhibited wound complications, infections, implant failures, or bone–implant interface failures.

**Conclusions:**

Fixation of the inferior ramus of a pubis-ischium ramus fracture based on conventional fixation of the anterior pelvic ring is mechanically superior in cadaveric Tile B pelvic injury and shows rapid recovery, good functional outcomes, and low incidence of complications.

## Introduction

Pelvic ring injuries occur frequently in cases of high-energy trauma and are associated with significant morbidity and mortality. Prior research has shown that early stabilization of the pelvis is critical for survival [1]. The continuity of the front pelvic ring is important for pelvic stability, as non-continuity leads to asymmetrical loads. The superior and inferior ramus of the pubis-ischium ramus are important parts of the anterior ring and act as biomechanical arches of the pelvis [2]. Studies suggest that anterior fixation enhances the stability of posterior fixation, and that an improper treatment of anterior injuries may lead to late failure of posterior fixation [3, 4]. However, the optimal strategy for anterior pelvic ring repair and fixation remains controversial [5]. Fixation of the superior ramus can be achieved through a variety of mechanisms, including intramedullary screws, INFIX, plates, and eternal fixation [6–9]. Currently, there is a paucity of data comparing the inferior ramus to the pubis-ischium ramus. While the anterior ring is so important for the stability of the pelvic ring, the importance of fixation of the puboischial ramus has not been verified by biomechanical results. Although percutaneous fixation for inferior ramus fracture nonunion has been reported in the literature, with early clinical success in small cases [10], the information presented in this prior study is limited, and the biomechanics of fixation remain unclear.

Therefore, the purpose of this study was to use a cadaveric model to evaluate the biomechanical properties of plate internal fixation of the inferior ramus of the pubis-ischium ramus and to observe the clinical outcomes of fixation of the fracture of the inferior ramus of the pubis-ischium ramus. Based on the conventional fixation of the anterior ring, we hypothesized that fixation of the inferior ramus of the pubis-ischium ramus would achieve both biomechanical and clinical advantages. Therefore, research on treating of anterior pelvic ring fractures is of great significance for guiding clinical treatment, promoting patients’ return to activities, and reducing the complications of anterior ring injuries.

## Results

### Biomechanical analysis

Under 500 N of loading, the average largest displacements in Group A were 0.26, 0.33, and 0.06 mm in L1, L2, and L3, respectively. The corresponding displacements were 0.61, 0.18, and 0.07 mm in Group B, and 0.11, 0.05, and 0.05 mm in Group C, respectively (Table 1). The displacements in Group C were significantly smaller at L1 and L2 than those in Groups A and B. The differences among the three groups at L3 were not statistically significant.

**Table 1:** Comparison of pelvic ring displacement distance (mm) under 500 N axial loading

Parameter	Group A	Group C	*P* value	Group A	Group B	*P* value	Group C	Group B	*P* value
L1	0.26 ± 0.05	0.11 ± 0.03	0.021	0.26 ± 0.05	0.61 ± 0.17	< 0.001	0.11 ± 0.03	0.61 ± 0.17	< 0.001
L2	0.33 ± 0.16	0.05 ± 0.02	< 0.001	0.33 ± 0.16	0.18 ± 0.04	0.015	0.05 ± 0.02	0.18 ± 0.04	0.043
L3	0.06 ± 0.02	0.05 ± 0.01	0.278	0.06 ± 0.02	0.07 ± 0.02	0.363	0.05 ± 0.01	0.07 ± 0.02	0.057

### Clinical results

The baseline characteristics of patients with no significant intergroup differences are shown in Table 2. Group D included 5 females and 8 males, with a mean age of 39.69 ± 9.15 years (range 26–52 years). The predominant injury mechanism was traffic accident injury (7 cases), followed by falling from a height (4 cases) and other injuries (2 cases). According to the Tile classification, the cohort included 6, 4, and 3 type B1, B2, and B3 fractures, respectively. Group E included 6 females and 7 males, with a mean age of 36.77 ± 9.52 years (range 19–51 years). The injury mechanisms were traffic accident injuries (8 cases), falling from a height (4 cases), and other injuries (1 case). According to the Tile classification, there were 5, 6, and 2 cases of type B1, B2, and B3 fractures. The same group of surgeons performed all surgeries.

**Table 2:** Patient demographics of two groups

Parameter	Age	Gender: male/female	Injury mechanism			Tile Classification		
			Traffic accident	Fall from height	Other	B1	B2	B3
Group D	39.69 ± 9.15	8/5	7	4	2	6	4	3
Group E	36.77 ± 9.52	7/6	8	4	1	5	6	2
*P*	0.433	0.691	0.819			0.708		

The mean follow-up time was 19.38 ± 4.19 months (range 13–26) in Group D and 17.92 ± 4.55 months (range 12–28) in Group E (Table 3). The mean time to surgery was 6.15 ± 3.13 days (range 2–13) in Group D and 6.54 ± 2.88 days (range 3–12) in Group E. The average operation time in Group D [131.54 ± 33.48 (range 83–196)] was significantly shorter than that in Group E [190.23 ± 30.33 (range 147–262)] (*P* < 0.001). No significant differences were observed in terms of blood loss. However, Group E [11.69 ± 5.95 (range 6–26)] had a shorter hospitalization time than Group D [17.92 ± 7.63 (range 8–32)] (*P* = 0.029).

**Table 3:** Operation-related indices and clinical data

Parameter	Follow-up time (month)	Time to surgery (day)	Operation time(min)	Blood loss (ml)	Hospitalization time (day
Group D	19.38±4.19	6.15±3.13	131.54±33.48	413.85±107.05	17.92±7.63
Group E	17.92±4.55	6.54±2.88	190.23±30.33	498.46±108.39	11.69±5.95
P	0.403	0.747	<0.001	0.057	0.029

**Fig. 1:**
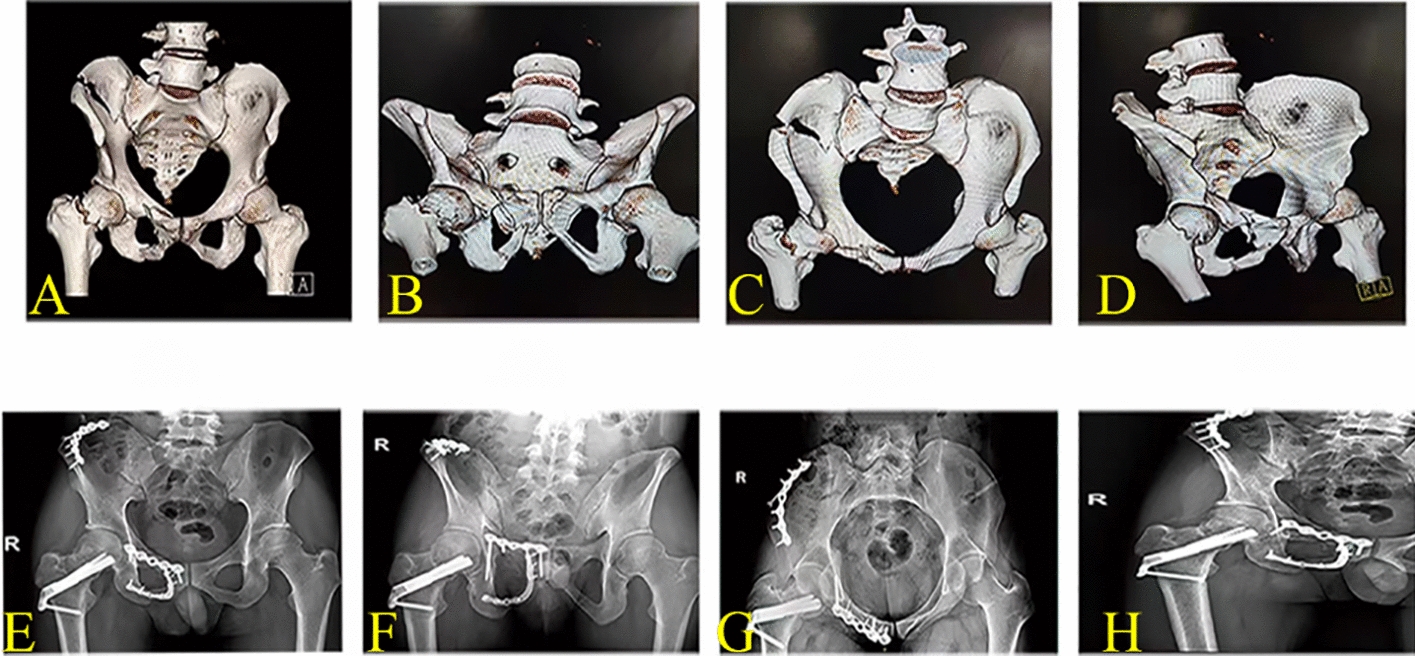
Typical case of the fixation of the superior and inferior ramus of pubis-ischium ramus fracture. **A**–**D** 3D reconstruction of preoperative CT images of the pelvis of a 32-year-old man with fractures of the right superior and inferior rami of the pubis-ischium ramus. **E**: anteroposterior view, **F**: outlet view, **G**: inlet view, **H**: obturator view, the postoperative X-ray of the male patient showing the male’s quality of fracture reduction was excellent

Clinical outcomes are presented in Table 4. The postoperative radiographic and functional outcomes are shown in Table 4. By referring to the relevant Matta radiological standards, we divided the quality of fracture reduction into four grades: excellent, good, fair and poor. In group D, 4 cases were excellent, 4 cases were good, 4 cases were fair, and 1 case was poor, while in Group E, there were 8 excellent cases (a typical case is shown in Fig. 1), 3 good cases, and 2 fair cases.

**Table 4:** Clinical outcomes

Parameter	Matta score					Weight-bearing (week)	Union time (week)	Majeed score	Sexual dysfunction rate
	Excellent	Good	Fair	Poor	Satisfactory rate				
Group D	4	4	4	1	8/13 (61.54%)	4.77 ± 2.09	16.55 ± 3.11	82.38 ± 8.81	7/13(53.85%)
Group E	8	3	2	0	11/13 (84.62%)	2.54 ± 1.45	13.23 ± 2.89	89.77 ± 7.27	2/13(15.38%)
*P*					0.185	0.004	0.013	0.028	0.039

The average time to weight-bearing exercise in Group E was 2.54 ± 1.45 (range 1–8) weeks, which was significantly earlier than that in Group D [4.77 ± 2.09 (range 76–100)] (*P* = 0.004).

The fractures achieved bony union in a mean duration of 16.55 ± 3.11 (range 12–22) weeks in Group D, excluding 2 cases of the inferior ramus of pubis-ischium ramus nonunion. All patients in Group E achieved bony union in a mean duration of 13.23 ± 2.89 (range 8–18) weeks, which was significantly shorter than that in Group D (*P* = 0.013). The average Majeed pelvic score in Group E [89.77 ± 7.27 (range 76–100)] was significantly higher than that in Group D [82.38 ± 8.81 (range 72–100)] (*P* = 0.028).

The incidence of sexual dysfunction was significantly higher in Group D than that in Group E (*P* = 0.039). Two patients developed non-union of the inferior ramus of pubis-ischium ramus fractures in Group D, while two patients had heterotopic ossification near the ischial tubercles in Group E, although neither experienced discomfort. No wound complications, infections, implant failures, or bone–implant interface failures occurred in any of the patients.

## Discussion

Restoring the anatomical structure and biomechanical stability of the pelvic ring, promoting early functional exercise, and accelerating bone healing are all important goals of surgical treatment of pelvic ring injuries. Studies have shown that 40% of pelvic stability is maintained by the anterior complex [11]. In a prior study, Liu et al. indicated that the stability of the posterior pelvic ring correspondingly increases with an increase in the stability of the anterior pelvic ring [12]. However, information regarding the role of the inferior ramus of the pubis-ischium as a stabilizer of the anterior pelvic ring is limited, and it is unclear whether repair of the inferior ramus of the pubis-ischium is beneficial. Furthermore, repair of the inferior ramus of the pubis-ischium ramus injury has not received adequate clinical attention. Therefore, this study was conducted to address the gap in scientific knowledge regarding the biomechanical capabilities of new techniques for treating injuries to the inferior ramus of the pubis-ischium ramus.

Studies have shown that patients with pelvic fracture displacement of < 1 cm have a good prognosis [13, 14]. Our results showed that when both the superior ramus and inferior ramus of the pubis-ischium ramus were fixed, the displacement of the posterior pelvic ring joint and the fracture of the anterior pelvic ring was less than 0.2 cm under loads of 500 N. These findings demonstrate that the superior ramus combined with the inferior ramus of the pubis-ischium ramus fixation can provide excellent biomechanical stability against anterior pelvic injuries. The biomechanical findings of this study indicate that repair and fixation of obturator ring injuries should be considered in clinical practice. Biomechanically, based on our data results, fixation of the superior and inferior ramus of pubis-ischium ramus may be an superior option for treating Tile B pelvic injuries.

Internal fixation of the inferior ramus of the pubis-ischium ramus through the lateral approach to the perineum is a safe and easy technique with few related complications. In the present study, we applied the lateral-perineal approach to the ischial tuberosity, which was located 4 cm lateral to the apex of the pubic arch point; this approach has also been reported previously [15].

Surgical indications for the inferior ramus of the pubis-ischium ramus are controversial; in general, fractures of the inferior ramus of the pubis-ischium ramus are treated as benign fractures and are considered to have little effect on healing of the pelvic ring; therefore, proper reduction and fixation of the fracture are neglected, resulting in complications. Currently, complications are the dominant indication for surgical treatment [16]. Persistent pain, sitting discomfort, lower limb discrepancies, and sexual dysfunction are all common complaints [16–19]. Furthermore, symptomatic nonunion or malunion of the inferior ramus of the pubis-ischium has aroused clinical concern [19–23]. Surgical treatment of the inferior ramus of the pubis-ischium ramus nonunion often requires bone grafting [17, 19], which is associated with an increased degree of medical trauma compared to initial fixation. Sexual dysfunction is a long-term complication of pelvic ring fractures that is often underestimated and unaddressed, resulting in feelings of shame and depression and reduced quality of life in patients [24, 25]. According to a review, the incidence of sexual dysfunction after pelvic fractures varies from 10.3 to 100% [26–28]. Several studies have shown that sexual dysfunction after pelvic fracture is related to multiple factors, including patient age, pelvic injury type, injury severity score, urethral injury, and pelvic floor soft tissue injury [10, 24, 29, 30]. Further investigation has indicated that sexual dysfunction is associated with pubic branch fractures and pubic symphysis injuries [26]. Nevertheless, whether repair of the inferior ramus in pubis-ischium ramus fractures has a positive effect on sexual function remains unelucidated. With an increasing understanding of the anatomy, biomechanics, and surgical techniques of pelvic injury, patients may benefit from recent efforts to prevent complications in the acute phase of fracture. In the present study, none of the patients experienced bone nonunion or malunion, and a low incidence (15.4%) of sexual dysfunction was observed, which may be related to the good reduction in the inferior ramus of the pubis-ischium ramus fractures.

Enhanced recovery after surgery (ERAS) is important in the management of limbs and joint injuries. However, ERAS in pelvic injury is a luxury. Based on the results of optimistic data on the biomechanics of the specimen, ERAS in pelvic injuries can be performed. In our clinical study, repair and internal fixation of the obturator ring (fixation of the superior ramus and inferior ramus of the pubis-ischium ramus) increased the steadiness of the pelvic ring and met the requirements of early weight-bearing exercises. During recovery, the patient’s ability to sit on a wheelchair, walk with crutches, and even have sexual intercourse were encouraged. In the typical case, although the young man can’t bear with weight as for complicated with femoral neck fracture, we actively performed the ERAS in the process of treatment. In the 8th week after surgery, he succeeded in regain satisfied intercourse, which is a great encouragement to patients with pelvic injuries. Therefore, repair and fixation of the inferior ramus of pubis-ischium ramus fractures should be given more attention and recommended for traumatic pelvic ring injuries.

Our study provides a foundation to promote the repair of the inferior ramus of the pubis-ischium ramus in patients with pelvic fractures. Furthermore, our study is the first to report the biomechanical stability of the inferior ramus of the pubis-ischium ramus, and our results showed increased stability of the pelvic ring when the superior and inferior rami of the pubis-ischium ramus were fixed. Nevertheless, this study has several limitations. First, the biomechanical tests were performed on a limited number of samples. Second, the specimens were not fresh, and the mechanical properties of the pelvis varied after formalin immersion. These limitations may have impeded the reliability of our results. However, the limited number of available cadaver specimens makes it difficult to perform tests on large samples. To solve this problem, prior researchers conducted multiple longitudinal load biomechanical tests using a single pelvic specimen [7]. Therefore, multiple groups of loading tests were conducted on the same specimen in the present study. In addition, the loss of normal physiological function of the muscle tissue in the specimens affected the data. Individuals with obesity experience loads of > 500 N in the pelvis; therefore, further investigations with additional loading tests are required. Third, we performed measurements using a Vernier caliper, which may yield inaccurate results; using a three-dimensional motion tracker, with characteristics of objectivity and high accuracy [5], would provide precise measurement data. Finally, the number of participants in this study is small, which may affect our clinical results. In the future, we will continue to promote this work, expand the number of cases, and conduct long-term follow-ups.

## Conclusion

When the inferior ramus of the pubis-ischium is fractured, it can be fixed using conventional anterior pelvic ring fixation procedures. However, in cases of Tile B pelvic ring injury, superior ramus combined with inferior ramus of the pubis-ischium fixation therapy can be employed as it has been found to provide higher biomechanical stability and better functional clinical results.

## Methods

### Cadaveric study

**Fig. 2:**
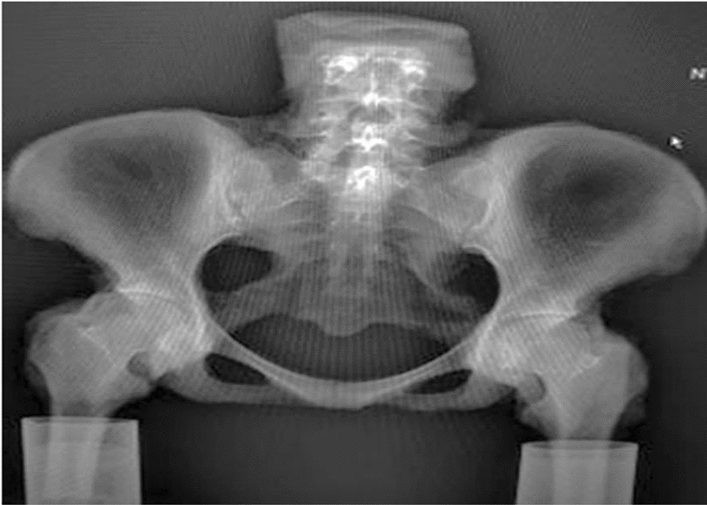
X-ray image of a pelvic specimen reveals the absence of pathological malformations or pelvic fractures

Six formalin-preserved human adult cadaveric pelvises (3 females and 3 males, mean age: 35.67 ± 12.94 years) were chosen for biomechanical testing at the Guangxi medical University following the attainment of consent from the donors’ families. For each specimen, a CT scan (Brilliance 64 CT, Philips Healthcare, Hamburg, Germany) was performed to determine the present volumetric bone mineral density (BMD). From the centers of each third lumbar vertebra, a voxel cube of 11 × 11 × 11 was segmented to determine trabecular volumetric BMD (Avizo version 5.1, Mercury Computer Systems, San Diego, USA). Based on the reference densities of the phantom, the average Hounsfeld unit value was linearly scaled. Based on the American College of Radiology guidelines, threshold values were used to differentiate between normal (> 120 mg/cm^3^), osteopenic (80–120 mg/cm^3^) and osteoporotic (80 mg/cm^3^) BMD [31]. The mean value of the BMD was 146.35 mg/cm^3^ (± 35.39 mg/cm^3^). Each specimen was free of pathological malformations or preexisting fractures of the pelvis. Approval was obtained from the Guangxi Medical University Ethics Committee [Number: 2022(KY-0145)]. Prior to biomechanical testing, the soft tissue of each pelvis was removed clearly, and the bilateral anterior and interosseous sacroiliac ligaments were disrupted. Preservation of posterior sacroiliac, sacrospinous, and sacrotuberous ligament integrity was ensured by retaining the L3–L5, sacrum, and 20 cm of the proximal femur. Pelvic specimens were observed with the naked eye and were subjected to radiography to rule out pathological malformations or preexisting fractures of the pelvis (Fig. 2).

### Pelvic injury model creation

**Fig. 3:**
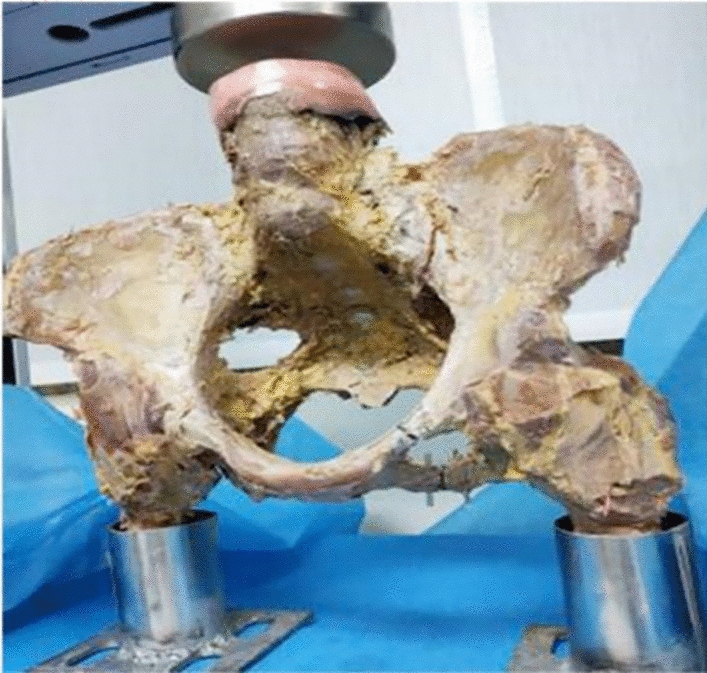
Tile B fracture cadaver model, each left superior ramus and the inferior ramus of the pubis-ischium was sawed vertically

**Fig. 4:**
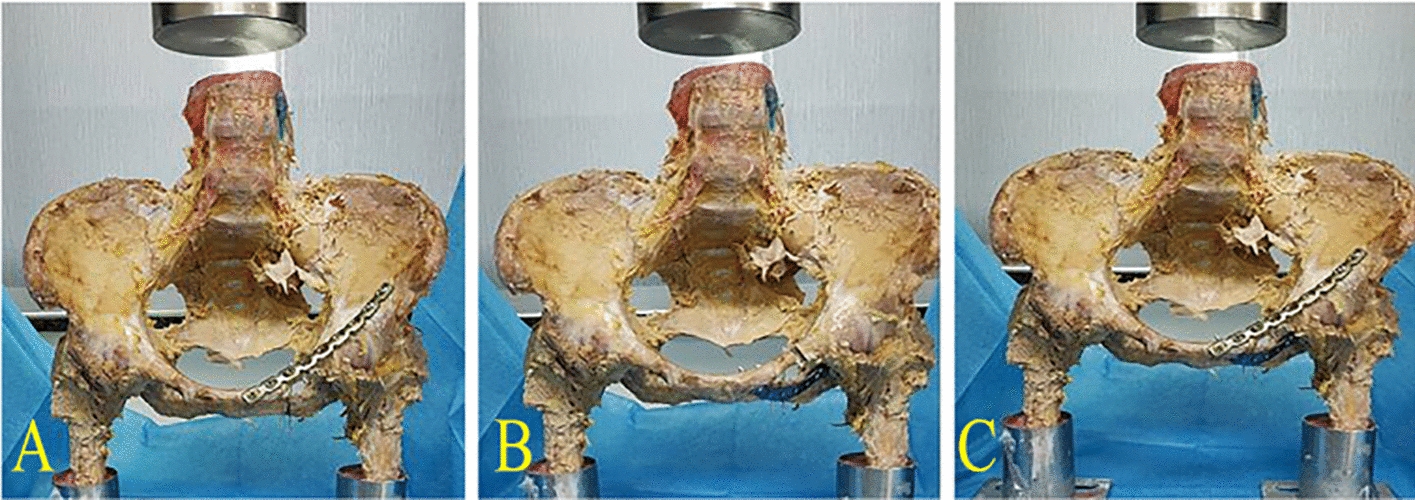
Biomechanical test model of three different reconstruction modes after pelvic injury. **A** fixation of superior ramus of pubis, **B** fixation of inferior ramus of pubis-ischium ramus, **C** fixation of superior and inferior ramus of pubis-ischium ramus

Models of Tile B pelvic injuries were created by sawing each left superior ramus and the inferior ramus of the pubis-ischium vertically with a saw (Fig. 3). Pelvises were separated into three groups based on the treatment (Fig. 4), as follows: the superior ramus was repaired with a 3.5 mm pelvic reconstruction plate (Group A); the inferior ramus of the pubis-ischium ramus underwent a 2.7 mm anatomical plate fixation (Group B), and the superior and inferior ramus of pubis-ischium ramus underwent two plates fixation (Group C). For this experiment, the Shandong Weigao Company (Weihai, China) provided all the implants, and they were implanted into the specimens by the same operator. We inserted two 1.5 mm K-wires into the vertical waterlines at the superior ramus (L1), inferior ramus of the pubis-ischium ramus (L2), and sacroiliac region (L3) to measure the distances between the two wires during the pressurization process (Fig. 3).

### Biomechanical testing

We performed all biomechanical experiments at the Guangxi Key Laboratory of Regenerative Medicine, Guangxi Medical University. The distal part of the femur and L3 vertebra were embedded and immobilized in a biomechanical testing machine (AGS-X, Shimadzu, Tokyo, Japan). All specimens were placed in a standing position and fixed. Axial compression of 10 N/s was exerted on the upper sacrum and sustained for a duration of 60 s until the load reached 500 N. The experiment was replicated a minimum of three times. The distance between two K-wires was measured three times using Vernier calipers (Germany MNT, China), and the mean value was calculated.

### Clinical research

We conducted a retrospective case study of patients who underwent surgery at our department between August 2019 and August 2022. A total of 26 patients with pubis-ischium ramus fractures of the superior and inferior ramus were included. The inclusion criteria for open reduction and internal fixation were: inferior ramus of the pubis-ischium ramus fractures with a displacement greater than 4 mm, separation displacement greater than 2 mm, and comminuted fractures. All patients agreed to participate and provided written informed consent prior to undergoing treatment. This study was approved by the institutional review board of our institute [Approval No.2022 (KY⁃0145)].

Before surgery, a complete routine preoperative examination, including blood biochemistry and pelvic CT was performed. Patient demographics, injury mechanisms, time to surgery, operative time, blood loss, time to weight-bearing rehabilitation exercises, duration of hospital stay, and postoperative complications were recorded.

Prior to the procedure, all patients were administered general anesthesia and positioned supine. Group D (8 males and 5 females) underwent plate fixation, and Group E (7 males and 6 females) underwent plate fixation of both the superior and inferior ramus of the pubis-ischium ramus. A lateral–perineal approach was used to perform fracture reduction and plate fixation of the inferior ramus of the pubis-ischium ramus injuries. In both groups, the adductor muscle was reconstructed before the incision was closed. Patients in Group D underwent routine non-weight-bearing functional rehabilitation exercises in bed. Patients in Group E were allowed to sit up or move in a wheelchair 1 day postoperatively, excluding those with combined injuries that required bed rest. Subsequently, weight-bearing function exercises were gradually performed after the X-ray showed satisfactory results and pain was tolerable.

Pelvic fracture reduction quality, fracture union rate, mean union time, and functional assessment results were used as clinical outcomes. Pelvic fracture reduction quality was evaluated by the Matta score [32]. All three pelvic radiographs showed a bridging callus defining fracture union. The Majeed score could be used to evaluate pelvic functionality [33].

### Statistical analysis

All statistical analyses were finished by SPSS 21.0 (IBM, Armonk, NY, USA). We described measurement data using the mean ± standard deviation (± *s*), and compared each group using one-way analysis. The enumeration-type data were analyzed by the Chi-square test. Statistical significance was defined as *P* < 0.05.

## Data Availability

This study asserts that all data have been publicly shared, and all pertinent research data has been presented in this publication or supplementary materials. For further information or inquiries, the corresponding author can be contacted and the corresponding author will provide them upon reasonable request. The authors of this manuscript declare no relationships with any companies, whose products or services may be related to the subject matter of the article.

## References

[1] Lawson MM, Peterson DF, Friess DM, Cook MR, Working ZM (2023). Delay of fixation increases 30-day complications and mortality in traumatic pelvic ring injuries. Eur J Orthop Surg Traumatol.

[2] Gamble JG, Simmons SC, Freedman M (1986). The symphysis pubis. anatomic and pathologic considerations. Clin Orthop Related Res.

[3] Bruce B, Reilly M, Sims S (2011). OTA highlight paper predicting future displacement of nonoperatively managed lateral compression sacral fractures: can it be done?. J Orthop Trauma.

[4] Choy WS, Kim KJ, Lee SK, Park HJ (2012). Anterior pelvic plating and sacroiliac joint fixation in unstable pelvic ring injuries. Yonsei Med J.

[5] Hempen EC, Wheatley BM, Schimoler PJ, Kharlamov A, Melvin PR, Miller MC (2022). A biomechanical comparison of superior ramus plating versus intramedullary screw fixation for unstable lateral compression pelvic ring injuries(,). Injury.

[6] McLachlin S, Lesieur M, Stephen D, Kreder H, Whyne C (2018). Biomechanical analysis of anterior ring fixation of the ramus in type C pelvis fractures. Eur J Trauma Emerg Surg.

[7] MacCormick L, Chen F, Gilbertson J, Khan S, Schroder L, Bechtold J (2019). A biomechanical study comparing minimally invasive anterior pelvic ring fixation techniques to external fixation. Injury.

[8] Berk T, Zderic I, Caspar J, Schwarzenberg P, Pastor T, Halvachizadeh S (2023). A novel implant for superior pubic ramus fracture fixation-development and a biomechanical feasibility study. Medicina.

[9] Berk T, Zderic I, Schwarzenberg P, Pastor T, Lesche F, Halvachizadeh S (2023). Evaluation of cannulated compression headless screws as an alternative implant for superior pubic ramus fracture fixation: a biomechanical study. Int Orthop.

[10] Sorensen M, Wessells H, Rivara F, Zonies D, Jurkovich G, Wang J (2008). Prevalence and predictors of sexual dysfunction 12 months after major trauma: a national study. J Trauma.

[11] Bi C, Wang Q, Nagelli C, Wu J, Wang Q, Wang J (2016). Treatment of unstable posterior pelvic ring fracture with pedicle screw-rod fixator versus locking compression plate: a comparative study. Med Sci Monit.

[12] Liu L, Zeng D, Fan S, Peng Y, Song H, Jin D (2021). Biomechanical study of Tile C3 pelvic fracture fixation using an anterior internal system combined with sacroiliac screws. J Orthop Surg Res.

[13] Tornetta P, Matta J (1996). Outcome of operatively treated unstable posterior pelvic ring disruptions. Clin Orthop Relat Res.

[14] Dujardin F, Hossenbaccus M, Duparc F, Biga N, Thomine J (1998). Long-term functional prognosis of posterior injuries in high-energy pelvic disruption. J Orthop Trauma.

[15] Wei L, Jianwen C, Shiting T, Jinmin Z, Zhi Y, Feng H (2022). Anatomic and clinical application of lateral-perineal approach for inferior ramus of pubis-ischium ramus. Chin J Orthop.

[16] Oransky M, Tortora M (2007). Nonunions and malunions after pelvic fractures: why they occur and what can be done?. Injury.

[17] Mears D, Velyvis J (2002). In situ fixation of pelvic nonunions following pathologic and insufficiency fractures. J Bone Joint Surg Am.

[18] Matta J (1996). Indications for anterior fixation of pelvic fractures. Clin Orthop Relat Res.

[19] Archdeacon M, Kuhlman G, Kazemi N (2010). Fellow’s corner: grand rounds from the university of cincinnati medical center–painful superior and inferior pubic rami nonunion. J Orthop Trauma.

[20] Templeman D, Simpson T, Matta J (2005). Surgical management of pelvic ring injuries. Instr Course Lect.

[21] Ebraheim N, Biyani A, Wong F (1998). Nonunion of pelvic fractures. J Trauma.

[22] Matta J, Dickson K, Markovich G (1996). Surgical treatment of pelvic nonunions and malunions. Clin Orthop Relat Res.

[23] Pennal G, Massiah K (1980). Nonunion and delayed union of fractures of the pelvis. Clin Orthop Relat Res.

[24] Ter-Grigorian A, Kasyan G, Pushkar D (2013). Urogenital disorders after pelvic ring injuries. Central Eur J Urol.

[25] Papadopoulos I, Kanakaris N, Bonovas S, Triantafillidis A, Garnavos C, Voros D (2006). Auditing 655 fatalities with pelvic fractures by autopsy as a basis to evaluate trauma care. J Am Coll Surg.

[26] Rovere G, Perna A, Meccariello L, De Mauro D, Smimmo A, Proietti L (2021). Epidemiology and aetiology of male and female sexual dysfunctions related to pelvic ring injuries: a systematic review. Int Orthop.

[27] Pohlemann T, Gänsslen A, Schellwald O, Culemann U, Tscherne H (1996). Outcome after pelvic ring injuries. Injury.

[28] Guan Y, Wendong S, Zhao S, Liu T, Liu Y, Zhang X (2015). The vascular and neurogenic factors associated with erectile dysfunction in patients after pelvic fractures. Inte Braz J Urol.

[29] Ceylan H, Kuyucu E, Erdem R, Polat G, Yιlmaz F, Gümüş B (2015). Does pelvic injury trigger erectile dysfunction in men?. Chin J traumatol = Zhonghua chuang shang za zhi.

[30] Vallier H, Cureton B, Schubeck D (2012). Pelvic ring injury is associated with sexual dysfunction in women. J Orthop Trauma.

[31] Fensky F, Weiser L, Sellenschloh K, Vollmer M, Hartel MJ, Morlock MM (2021). Biomechanical analysis of anterior pelvic ring fractures with intact peripelvic soft tissues: a cadaveric study. Eur J Trauma Emerg Surg.

[32] Matta J, Tornetta P (1996). Internal fixation of unstable pelvic ring injuries. Clin Orthop Relat Res.

[33] Majeed S (1989). Grading the outcome of pelvic fractures. J Bone Joint Surg Br Vol.

